# Association between coffee consumption and periodontal diseases: a systematic review and meta-analysis

**DOI:** 10.1186/s12903-022-02310-2

**Published:** 2022-07-05

**Authors:** Yeonjae Rhee, Yongjun Choi, Jeongmin Park, Hae Ryoun Park, Kihun Kim, Yun Hak Kim

**Affiliations:** 1 School of Dentistry, Pusan National University, Yangsan, 50610 Republic of Korea; 2 Department of Oral Pathology, School of Dentistry, Pusan National University, Yangsan, 50610 Republic of Korea; 3 Dental and Life Science Institute, School of Dentistry, Pusan National University, Yangsan, 50610 Republic of Korea; 4 Periodontal Disease Signaling Network Research Center (MRC), School of Dentistry, Dental Research Institute, Pusan National University, Yangsan, 50610 Republic of Korea; 5 Department of Occupational and Environmental Medicine, Kosin University Gospel Hospital, Busan, Republic of Korea; 6 Department of Biomedical Informatics, School of Medicine, Pusan National University, Yangsan, 50610 Republic of Korea; 7 Department of Anatomy, School of Medicine, Pusan National University, Yangsan, 50610 Republic of Korea

**Keywords:** Coffee, Periodontitis, tooth loss, Observational study, Systematic review, Meta-analysis

## Abstract

**Background:**

Several studies have demonstrated association between coffee consumption and periodontal diseases. However, no systematic review and meta-analysis was performed. Therefore, we performed a systematic review and meta-analysis to evaluate the association between coffee intake and periodontitis.

**Methods:**

We defined PICO statement as “Do coffee drinkers have a higher association of periodontitis or tooth loss than non-coffee drinkers?”. We searched for articles using the Embase and Medline databases. The odds ratio was used as an effect measure to evaluate the association between coffee and periodontitis We divided coffee intake doses into three groups: no intake (≤ 0.03 cups/day), low intake (0.03 < x < 1 cups/day), and high intake (≥ 1 cup/day). Cohort and cross-sectional studies were eligible for inclusion in this study. The Newcastle–Ottawa scale was used to qualitatively assess the risk of bias. The degree of heterogeneity between studies was quantified using I^2^ statistics.

**Results:**

Six articles were analysed, including two cohort studies and four cross-sectional studies. The pooled unadjusted odds ratios of periodontitis were 1.14 (0.93–1.39), 1.05 (0.73–1.52), 1.03 (0.91–1.16) and 1.10 (0.84–1.45) in the 4 meta-analyses (coffee drinker vs. non-coffee drinker, high intake vs. low intake, low intake vs. no intake, high intake vs. no intake), respectively.

**Conclusion:**

This is the first meta-analysis to investigate the relationship between coffee consumption and periodontitis. There was no relationship between coffee consumption and periodontitis. Further studies are required to assess whether a relationship between coffee consumption and periodontitis exists or not.

*PROSPERO registration number*: CRD42022301341.

**Supplementary Information:**

The online version contains supplementary material available at 10.1186/s12903-022-02310-2.

## Introduction

Coffee is one of the most consumed beverages in the world. Its consumption is second in the beverage market after water consumption [1]. It has been reported to have many positive effects on human health [2]. Caffeine, a component of coffee, exerts antioxidant and anti-inflammatory effects. Furthermore, one study has suggested that chlorogenic acid from coffee has potent chemopreventive effects [3].

Many studies have investigated the relationship between coffee consumption and systemic diseases. Coffee has been proven to lower the risk of Alzheimer’s disease, Parkinson's disease, type 2 diabetes, liver cancer, and heart attack [4]. In contrast, it has been reported that coffee can also be harmful since it causes insomnia, restlessness, and high blood pressure [5–7].

Periodontal disease is an chronic oral inflammation and infection that damages the supporting tissues around the teeth [8]. Cytokines produced during periodontitis may enter the systemic circulation and can lead to other health complications [9]. It is highly prevalent worldwide and is one of two major oral diseases [10]. Periodontitis is closely associated with lifestyle, especially intake of medications and alcohol [11–13]. It has also been linked to systemic disease such as cancer and respiratory infections [14, 15]. Severe periodontitis also caused oral diseases and tooth loss [16, 17]. Hence, we decided to investigate the influence of coffee on the periodontitis.

Several papers have been published containing information on the association between coffee and periodontitis [18–27]. According to Han et al. (2016), the consumption of two or more cups of coffee each day may be considered as risk factor for periodontitis [22]. Meanwhile, Hong et al. (2021) reported no statistically significant association between coffee and periodontitis [19]. There is no currently systematic review and meta-analysis that comprehensively analysed association between coffee consumption and periodontal diseases. Therefore, we performed a systematic review and meta-analysis to evaluate this association.

## Material and methods

### Eligibility criteria

We defined PICO statement as “Do coffee drinkers have a higher association of periodontitis or tooth loss than non-coffee drinkers?”. All observational studies with detailed information on coffee consumption and periodontal diseases were eligible for inclusion in this study. Only articles published in English were included; however, publication year was not restricted. We also excluded non-human articles and non-articles type papers from the search.

### Information sources and search strategy

This meta-analysis was conducted in accordance with the PRISMA guidelines [28]. We searched for articles using the Embase and Medline databases published until January 3, 2022. The search strategy was as follows: (coffee:ab,ti OR caffeine:ab,ti OR caffeinated:ab,ti) AND (periodontitis:ab,ti OR periodontal disease:ab,ti OR periodontal inflammation:ab,ti OR gum disease:ab,ti OR gum inflammation:ab,ti OR gingivitis:ab,ti OR periodontitis:ab,ti OR paradentitis:ab,ti OR oral health:ab,ti OR oral disease:ab,ti OR oral hygiene:ab,ti OR tooth loss:ab,ti OR missing teeth:ab,ti) AND (risk:ab,ti OR ratio:ab,ti OR prevalence:ab,ti OR incidence:ab,ti OR outcome:ab,ti OR prognosis:ab,ti OR hazard:ab,ti OR odds:ab,ti OR morbidity:ab,ti) AND ([article]/lim OR [article in press]lim) ANd {English]/lim AND [humans]lim.

### Selection process

Two authors (YR and YC) independently selected suitable papers from the screened records and evaluated the eligibility of the papers. The same authors searched grey literatures by combining words included in the search strategy using Google and Google Scalar. In evaluating the papers' eligibility, disagreements were resolved through discussion by the authors.

### Data collection process and data items

We extracted the following data during the screening phase: title, abstract, author name, publication year, publication type, article language, and summary language. Through a full-text assessment, the name of the disease, study year and region, number of samples, age, sex ratio, and effect sizes were added. We included studies that presented the number of samples or effect sizes according to our dose criteria. Papers containing unavailable data were excluded if they did not match the dose criteria set in this study.

### Study risk of bias assessment

The Newcastle–Ottawa scale was used to qualitatively assess the risk of bias in the cohort studies [29]. For cross-sectional studies, the adapted version of the Newcastle–Ottawa scale presented by Herzog et al. was used [30]. The assessment tools are presented in the Additional file 1: Tables. We assessed the risk of bias in the included studies and verified the quality of evidence.

### Effect measure

The odds ratio and 95% confidence intervals (CIs) were used to evaluate the association between coffee and periodontal diseases. For articles that did not represent the odds ratio, we calculated the odds ratio using the number of samples. The odds ratio was followed by unadjusted values and 95% CIs.

### Synthesis methods

Data were shown as crude odds ratios (ORs) with 95% CIs. The overall degree of heterogeneity between studies was quantified using I^2^ statistics [31]. We used the random effect model because the heterogeneity of all results was more than 50%. Review Manager 5.4 software was used to synthesize the results. First, we compared coffee drinkers with non-coffee drinkers. Second, we compared high- and low-intake drinkers. Third, we compared low-intake drinkers with non-coffee drinkers. Fourth, we compared high-intake drinkers to non-coffee drinkers. Additionally, we analysed the relationship between coffee consumption and tooth loss. In the case of tooth loss, we compared coffee drinkers with non-coffee drinkers.

### Certainty assessment

The GRADE method was used to assess the quality of evidence for the main outcome as high, moderate, low, or very low based on five required domain and three additional domains [32, 33].

## Results

### Study selection and characteristics

**Fig. 1:**
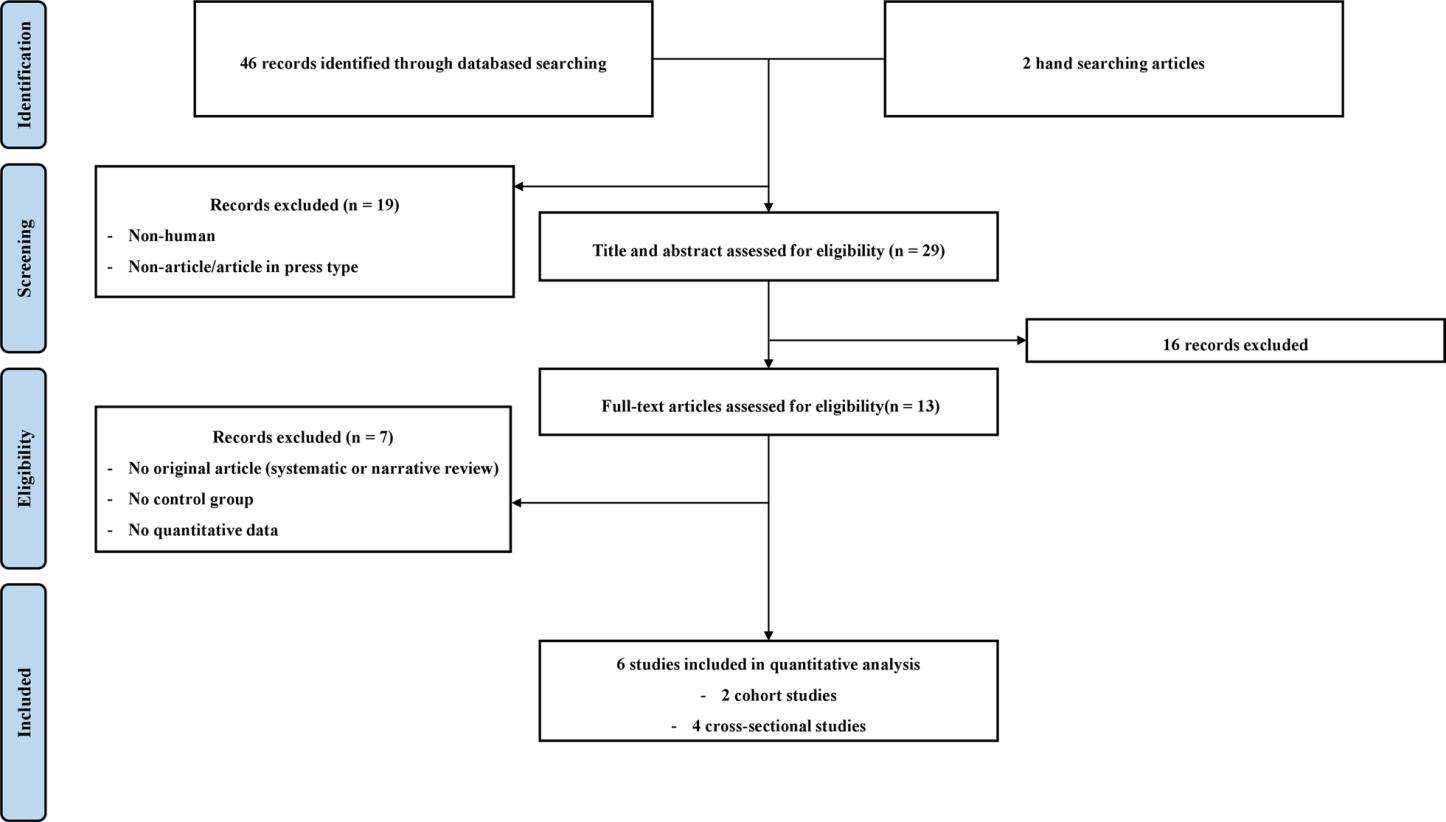
PRISMA flowchart

A total of 46 records were identified based on the search terms, and 2 hand searching articles were additionally identified. Nineteen non-human subjects and non-article type papers were excluded. First, 29 studies were screened based on their titles and abstracts. Second, 16 articles that were unrelated to our study topic were excluded. Third, a full-text review was conducted on the remaining 13 articles. We excluded five articles based on the following criteria: no original article (systematic or narrative review), no control group articles, and no quantitative data. Finally, six articles were included (Fig. 1). These included two cohort studies and four cross-sectional studies. The characteristics of the included studies are shown in Table 1.

**Table 1:** Characteristics of included studies

References	Study design	Country	Year of study	No. of participants	Male/female	Definition of Coffee intake	Definition of periodontal disease	Comments
Hong et al. [19]	Cohort	Korea	2004–2016	134,855	47,123/87,732	No drink mild drink (one time a month through six times a week) Heavy drink (one or more times a day)	Periodontitis—Yes or no (based-on questionnaire)	Coffee intake and periodontitis are not significant
Abbass et al. [20]	Cross-sectional	Egypt	2018	343	139/204	≤ 2 times/week 3–6 times/week 1–6 times/day	Periodontitis—Clinical and radiographic case identification was performed by trained examiners according to the latest classification of periodontal diseases	Caffeinated drinks were shown to have a positive correlation with periodontitis
Han et al. [22]	Cross-sectional	Korea	2008–2010	16,730	6,716/10,014	≤ Once per month Once per month < x ≤ 3 times per week Three times per week < x ≤ 6 times per week Once per day Twice per day Three or more per day	Periodontitis—Yes or no based-on community periodontal index score	Consumption of coffee may be considered an independent risk indicator of periodontal disease in Korean male adults
Zuccarello et al. [23]	Cohort	Italy	–	206	98/108	Yes or no information obtained by participants	Chronic periodontitis—The diagnosis was based on the guidelines of the International Workshop for the Classification of Periodontal Disease and Conditions	No association was found between chronic periodontitis and lifestyles (coffee). Only familiarity showed a strong correlation
Koyama et al. [40]	Cross-sectional	Japan	2006	25,078	12,019/13,059	< 1 cups/day 1–2 cups/day 3–4 cups/day 5 ≥ cups/day	Tooth loss—Yes (< 20 teeth) / no (≥ 20 teeth)	People who consumed more cups of coffee had a lower number of teeth
Tanaka et al. [41]	Cross-sectional	Japan	2002–2003	1,002	0/1,002	< 1 time/week 1–6 times/week 1 + time/day	Tooth loss—Yes (+ 1 extraction teeth) / no (no extraction teeth)	Coffee consumption was independently associated with an increased prevalence of tooth loss

### Synthesis of the results

**Fig. 2:**
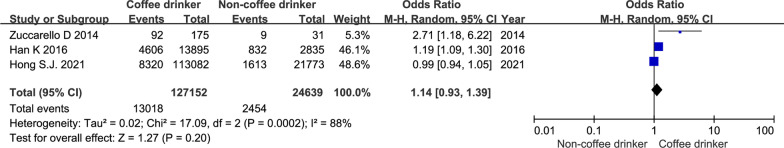
Forest plot for the association between coffee consumption and periodontitis

4 studies were used to evaluate the association between coffee and periodontitis. The pooled unadjusted odds ratios of periodontitis were 1.14 (95% CI 0.93–1.39; I^2^, 88%) (Fig. 2), 1.05 (95% CI 0.73–1.52; I^2^, 97%), 1.03 (95% CI 0.91–1.16; I^2^, 72%) and 1.10 (95% CI 0.84–1.45; I^2^, 96%) in the 4 meta-analyses (coffee drinker vs non-coffee drinker, high intake vs low intake, low intake vs no intake, high intake vs no intake), respectively (Table 2).

**Table 2:** The number of subjects included in each paper and association between the amount of coffee consumption and periodontitis

Study	Events	Total	Events	Total	Odds ratio			Heterogeneity		Test for overall effect	
					Weight	M-H, Random, 95% CI	Year	Chi^2^	I^2^	Z	p
	**Coffee drinker**		**Non-coffee drinker**								
Zuccarello D. et al	92	175	9	31	5.3%	2.71 [1.18, 6.22]	2014	17.09	88%	1.27	0.20
Han K. et al	4606	13,895	832	2835	46.1%	1.19 [1.09, 1.30]	2016				
Hong S.J. et al	8320	113,082	1613	21,773	48.6%	0.99 [0.94, 1.05]	2021				
Overall	13,018	127,152	2454	24,639	100.0%	1.14 [0.93, 1.39]					
	**High Intake**		**Low Intake**								
Han K. et al	3666	10,598	940	3297	44.3%	1.33 [1.22, 1.44]	2016	63.45	97%	0.26	0.79
Abbass M.M.S. et al	256	286	52	57	10.6%	0.82 [0.30, 2.21]	2020				
Hong S.J. et al	6121	85,597	2199	27,485	45.0%	0.89 [0.84, 0.93]	2021				
Overall	10,043	96,481	3191	30,839	100.0%	1.05 [0.73, 1.52]					
	**Low Intake**		**No Intake**								
Han K. et al	940	3297	832	2835	43.4%	0.96 [0.86, 1.07]	2016	3.54	72%	0.48	0.63
Hong S.J. et al	2199	27,485	1613	21,773	56.6%	1.09 [1.02, 1.16]	2021				
Overall	3139	30,782	2445	24,608	100.0%	1.03 [0.91, 1.16]					
	**High Intake**		**No Intake**								
Han K. et al	3666	10,598	832	2835	49.2%	1.27 [1.16, 1.39]	2016	26.39	96%	0.71	0.48
Hong S.J. et al	6121	85,597	1613	21,773	50.8%	0.96 [0.91, 1.02]	2021				
Overall	9787	96,195	2445	24,608	100.0%	1.10 [0.84, 1.45]					

**Fig. 3:**
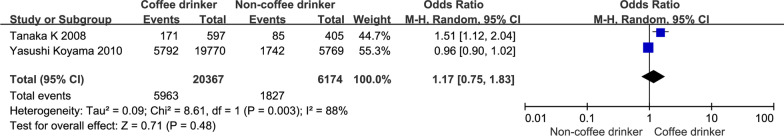
Forest plot for the association between coffee consumption and tooth loss

2 studies were used to evaluate the association between coffee and periodontitis. As severe periodontitis often leads to tooth loss, we also analysed two studies that included information on tooth loss cases depending on the consumption of coffee (Table 3). The odds ratio was 1.17 (95% CI 0.75–1.83; I^2^, 88%) (Fig. 3). Similar to the above results, the association between tooth loss and coffee intake was not statistically significant.

**Table 3:** The number of subjects included in each paper and association between coffee consumption and tooth loss

Study	Events	Total	Events	Total	Odds ratio			Heterogeneity		Test for overall effect	
					Weight	M-H, Random, 95% CI	Year	Chi^2^	I^2^	Z	p
	**Coffee drinker**		**Non-coffee drinker**								
Tanaka K. et al	171	597	85	405	44.7%	1.51 [1.12, 2.04]	2008	8.61	88%	0.71	0.48
Koyama Y. et al	5792	19,770	1742	5769	55.3%	0.96 [0.90, 1.02]	2010				
Overall	5963	20,367	1827	6174	100.0%	1.17 [0.75, 1.83]

### Risk of bias in studies

Risk of bias was evaluated for 2 cohort studies and 4 cross-sectional studies. The risk of bias of 2 cohort studies and 3 cross-sectional studies was rated as 'good', and 1 cross-sectional study was evaluated as ‘satisfactory’ (Additional file 1).

### Certainty assessment

The quality of evidence was evaluated for the main outcome. The quality of evidence was assessed to be very low, based on the GRADE method (Table 4).

**Table 4:** GRADE method for the primary outcome

Outcome	Quality assessment								
	Required domains					Additional domains			Grade
	Study limitations	Consistency	Directness of evidence	Precision	Reporting bias	Dose–response association	Plausible confounding that would decrease observed effect	Strength of association (magnitude of effect)	
Periodontitis	High^a^	Inconsistent^b^	Indirect	Precise^c^	Unevaluable^d^	Undetected	Present^e^	Weak^f^	⨁◯◯◯ Very low
Tooth loss	High^a^	Inconsistent^b^	Indirect	Precise^c^	Unevaluable^d^	Undetected	Present^e^	Weak^f^	⨁◯◯◯ Very low

^**a**^All-included studies are observational design

^b^Considerable heterogeneity (I^2^ > 50%)

^**c**^Sample size over 4000

^d^Due to small number of included studies

^**e**^All-included studies are observational design, and all analyses were based on unadjusted estimates

^f^OR < 2.0

## Discussion

Coffee intake was not found to be associated with periodontitis or tooth loss. This is the first meta-analysis to investigate the association between coffee consumption and periodontal diseases.

The effect of coffee on chronic inflammatory diseases remains controversial. C-reactive protein (CRP) is a known biomarker for inflammation [34]. One study found no association between coffee consumption and CRP [35]. On the contrary, some studies have suggested that coffee is beneficial for chronic inflammation [36]. Periodontitis is induced by an imbalance between the oral microbiota and the immune system, and coffee enhances the richness of the oral microbiome [9, 37]. Previous studies have shown that coffee consumption is not related to periodontitis; however, it is necessary to further analyse this relationship through a large-scale cohort study.

Periodontitis is an inflammatory condition where immune cells produce cytokines, such as IL-1 and IL-6. These factors activate osteoclasts, which destroy the alveolar bone, and inhibit bone forming osteoblasts. In addition, periodontal pathogenic bacteria directly inhibit osteoblasts and cause alveolar bone destruction, leading to tooth loss [38, 39]. According to our result, odds ratio for coffee intake and tooth loss was 1.17. Although we analysed only two cross-sectional studies, one of them had a significant result. Therefore, further large-scale cohort studies are required.

Our study had several limitations. First, the number of included studies was small. In addition, the criteria for dividing the dose levels varied in each study included in the analysis. Therefore, we had to create a new standard that could be applied to all six studies to categorise coffee doses. During this process, we assumed ‘less than 1 time per month (= less than 0.03 times per day)’ as ‘no coffee intake’. In addition, the exact quantity of coffee intake measured in these studies was not clear. The unit for the dose measurement was often indicated as ‘times’ or ‘cups’, and the exact amount of coffee or espresso shots included in each ‘time’ or ‘cup’ was not mentioned. Finally, there was variability among the definitions of periodontitis (e.g., clinical-based, self-reported questionnaire).

Despite these limitations, our study has several strengths. Our analysis was not on coffee consumption only, but was also done by the level of doses (low or high). Our odds ratios were consistent across all circumstances. Finally, as shown in the Additional file 1: Tables, most of included studies in our analysis were high quality.

## Conclusion

In conclusion, there was no association between coffee consumption and periodontal diseases according to our study There is a lack of research on coffee and periodontal disease, and each paper defines coffee consumption and periodontal disease differently. Thus, it is important to interpret the results carefully. Future research needs to be conducted with a large number of subjects, including a more detailed definition of coffee consumption and periodontal disease.

## Supplementary Information

**Additional file 1**. We assessed the risk of bias in the included studies and verified the quality of evidence.

## Data Availability

All data generated or analysed during this study are included in this published article.
