# Supplementary material for: Association between coffee consumption and periodontal diseases: a systematic review and meta-analysis
Source: BMC Oral Health. 2022 Jul 5;22:272. doi: 10.1186/s12903-022-02310-2 (PMC9258107; doi:10.1186/s12903-022-02310-2)
Supplement: Supplementary file 1 — Additional file 1. We assessed the risk of bias in the included studies and verified the quality of evidence. [file 12903_2022_2310_MOESM1_ESM.docx]

| **Study** | **Selection** | | | | **Comparability of cohorts on the basis of the design or analysis** | **Outcome** | | | **Total** | **Assessment** |
| --- | --- | --- | --- | --- | --- | --- | --- | --- | --- | --- |
|  | **Representativeness of exposed cohort** | **selection of nonexposed cohort** | **ascertainment of cohort** | **Demonstration that outcome of interest was not present at start of study** |  | **Assessment of outcome** | **Was follow-up long enough for outcomes to occur** | **Adequacy of follow up of cohorts** |  |  |
| Hong S.J. et al., 2021, Korea |  | ★ | ★ | ★ | ★★ | ★ | ★ | ★ | 8 | Good |
| Zuccarello D. et al., 2014, Italy | ★ | ★ | ★ |  | ★★ | ★ |  | ★ | 7 | Good |

Supplementary Table 1. Assessment (Cohort studies)

| **Source** | **Selection** | | | | **Comparability based on design and analysis** | **Outcome** | | **Total** | **Assessment** |
| --- | --- | --- | --- | --- | --- | --- | --- | --- | --- |
|  | **Representativeness of the sample** | **Sample size** | **Non-respondents** | **Ascertainment of the exposure** |  | **Assessment of outcome** | **Statistical test** |  |  |
| Abbass M.M.S. et al., 2020, Egypt | ★ | ★ | ★ | ★★ | ★★ | ★★ | ★ | 10 | Good |
| Han K. et al.,  2016, Korea | ★ |  | ★ | ★★ | ★★ | ★★ | ★ | 9 | Good |
| Tanaka K. et al., 2008, Japan |  |  | ★ | ★★ | ★★ | ★★ | ★ | 8 | Good |
| Koyama Y. et al., 2010, Japan |  |  |  | ★★ | ★★ | ★ | ★ | 6 | Satisfactory |

Supplementary Table 2. Assessment (cross-sectional studies).
